# Adenosine and Metabotropic Glutamate Receptors Are Present in Blood Serum and Exosomes from SAMP8 Mice: Modulation by Aging and Resveratrol

**DOI:** 10.3390/cells9071628

**Published:** 2020-07-07

**Authors:** Alejandro Sánchez-Melgar, José Luis Albasanz, Christian Griñán-Ferré, Mercè Pallàs, Mairena Martín

**Affiliations:** 1 Department of Inorganic, Organic and Biochemistry, Faculty of Chemical and Technological Sciences, School of Medicine of Ciudad Real, Regional Center of Biomedical Research (CRIB), University of Castilla- La Mancha (UCLM), 13071 Ciudad Real, Spain; alejandro.sanchez@uclm.es (A.S.-M.); mairena.martin@uclm.es (M.M.); 2 Department of Pharmacology and Therapeutic Chemistry, Faculty of Pharmacy and Food Sciences, Institute of Neuroscience, University of Barcelona, 08028 Barcelona, Spain; christian.grinan@ub.edu (C.G.-F.); pallas@ub.edu (M.P.)

**Keywords:** G-protein coupled receptors, adenosine receptors, metabotropic glutamate receptors, exosomes, blood serum, resveratrol, Alzheimer’s disease, SAMP8 mice

## Abstract

Adenosine (ARs) and metabotropic glutamate receptors (mGluRs) are G-protein coupled receptors (GPCRs) that are modulated in the brain of SAMP8 mice, an animal model of Alzheimer’s disease (AD). In the present work, it is shown the presence of ARs and mGluRs in blood serum and derived exosomes from SAMP8 mice as well as its possible modulation by aging and resveratrol (RSV) consumption. In blood serum, adenosine A_1_ and A_2A_ receptors remained unaltered from 5 to 7 months of age. However, an age-related decrease in adenosine level was observed, while 5′-Nucleotidase activity was not modulated. Regarding the glutamatergic system, it was observed a decrease in mGluR_5_ density and glutamate levels in older mice. In addition, dietary RSV supplementation caused an age-dependent modulation in both adenosinergic and glutamatergic systems. These GPCRs were also found in blood serum-derived exosomes, which might suggest that these receptors could be released into circulation via exosomes. Interestingly, changes elicited by age and RSV supplementation on mGluR_5_ density, and adenosine and glutamate levels were similar to that detected in whole-brain. Therefore, we might suggest that the quantification of these receptors, and their corresponding endogenous ligands, in blood serum could have predictive value for early diagnosis in combination with other distinctive hallmarks of AD.

## 1. Introduction

Alzheimer’s disease (AD) is the most common neurodegenerative disease, with around 50 million people affected. It is expected that by 2050 the incidence of AD will triplicate worldwide [1]. Unfortunately, when AD is diagnosed it is too late to reverse the neuronal death and cognitive decline. Therefore, it is necessary to find new biomarkers for early diagnosis to get preventive treatment. In the last years, imaging techniques such as positron emission tomography (PET) have provided useful information to aid in diagnosis [2], but this information alone appears to be inconclusive. Extensive studies on biomarkers of AD in cerebrospinal fluid (CSF) have evidenced the presence of amyloid-β (Aβ) peptide [3,4], Tau as well as phosphorylated Tau (p-Tau) [5,6], and even a potential association with apolipoprotein E (APOE) ε4 allele [3,7]. However, CSF analysis of AD biomarkers is not very useful as a routine tool for early diagnosis of AD as it requires a highly invasive lumbar puncture, and the results obtained seem to be also inconclusive [8]. In the last decade, the blood-based biomarkers are getting the attention of researchers due to it is a far-less invasive method [9]. Likewise, it has been reported the presence in blood of some potential biomarkers ranging from oxidative stress processes, mitochondrial dysfunction, neuronal injury, and pro-inflammatory cytokines, even Aβ, all of them distinctive hallmarks in AD [10,11]. However, the quantification of Aβ peptide density in peripheral blood seems to be highly variable and not useful for blood-based AD diagnosis [12].

Adenosine is a nucleoside widespread in the body that mainly operates through four adenosine receptors (ARs) and whose levels can be fine-tune regulated by its converting enzyme 5′-Nucleotidase [13]. ARs belong to G-protein coupled receptors (GPCRs) family and have been classified into A_1_, A_2A_, A_2B_, and A_3_ [14,15]. In the brain, adenosine is widely known as a modulator of neurotransmission, displaying a crucial role under physiological and pathological conditions [16,17]. Both A_1_ and A_2A_ receptors are the most abundant ARs in the central nervous system (CNS), and its role in neurodegenerative diseases, including AD, has been intensely investigated [17]. It has been reported that A_1_ and A_2A_ were altered in the frontal cortex [18], as well as adenosine level and 5′-nucleotidase activity in several cortical areas from post-mortem human brain of AD patients [19].

Glutamate is the main excitatory neurotransmitter in the CNS, whose action is mediated through ionotropic and metabotropic receptors [20]. The physiological role of this neurotransmitter is essential in synaptic transmission, neuronal plasticity, learning, and memory. Nevertheless, excessive concentration of glutamate may trigger ionotropic receptors activation that leads to excitotoxicity, neuronal dysfunction, and subsequent neuronal death. Indeed, glutamate-mediated excitotoxicity has been related to several neurological and neurodegenerative diseases including AD [21]. Interestingly, metabotropic glutamate 5 receptor (mGluR_5_), which belongs to the GPCR family, has been postulated as a potential therapeutic target since it was reported that amyloid-β (Aβ) directly interacts with mGluR_5_ [22]. In line with this, the group I mGluRs (mGluR_1_ and mGluR_5_) was found to be altered in the frontal cortex from the post-mortem human brain of AD patients [23].

Resveratrol (RSV) has been considered as an anti-aging molecule with several beneficial properties for health ranging from cardio- [24], and neuroprotection [25] as well as an antitumoral [26,27] and immunoregulatory [28] action, among others. Recently, it has been described the modulatory effect of RSV on adenosinergic [29] and glutamatergic systems [30] in the brain of SAMP8 mice from 5 and 7 months of age.

It is well established that receptors such as GPCRs or ionotropic receptors are mainly located into the plasma membrane, except for some receptors that are present in intracellular compartments (e.g., estrogen receptors in the nucleus). However, it has been recently reported the presence of receptors in circulation. The biological significance of those results remains to be clarified but the authors reported a potential correlation with some particular diseases [31,32], suggesting a predictive value in diagnosis.

Now, we show for the first time the presence of ARs and mGluRs, as well as their corresponding endogenous ligands, in blood serum and derived exosomes in SAMP8 mice. Moreover, some components from both adenosinergic and glutamatergic systems seem to be strongly affected by RSV supplementation. Intriguingly, changes in adenosine and glutamate levels, and mGluR_5_ density associated with aging detected here mimics those previously reported by our group in the brain from SAMP8 mice.

## 2. Materials and Methods

### 2.1. Animals and Resveratrol Diet

**Scheme 1:**
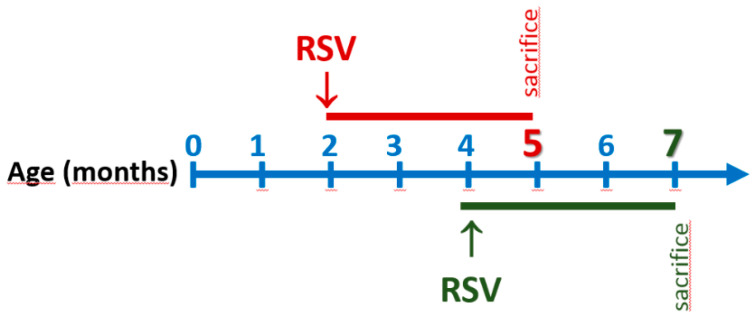
Resveratrol (RSV) treatment schedule followed in the present work.

A total of 26 male SAMP8 mice from 5 and 7 months-old (mo) were used for this study. Mice received a standard diet (2018 Teklad Global 18% Protein Rodent Maintenance Diet, ENVIGO, Barcelona, Spain) or the same diet supplemented with trans-resveratrol (RSV) (1 g/kg, Mega Resveratrol, Candlewood Stars, Inc., Danbury, CT, USA), starting from the weaning or 4 mo for 5 and 7 mo mice, respectively (Scheme 1). All the mice had food and water ad libitum and were kept in standard conditions of temperature (22 ± 2 °C) and 12:12-h light-dark cycles (300 lux/0 lux). There were no diet intake related differences (i.e., diet taste preference). There were not significant changes in food intake between groups. Food intake was routinely controlled, and revealed that, by mean, each animal eats 5 g of chow by day. Therefore, this RSV supplementation results in a daily dose of 160 mg/kg (body weight). All experimental procedures involving animals were performed followed by standard ethical guidelines European Communities Council Directive 86/609/EEC and by the Institutional Animal Care and Use Committee of the University of Barcelona (670/14/8102, approved at 11/14/2014) and by Generalitat de Catalunya (10291, approved 1/28/2018). All efforts were made to minimize the number of mice used and their suffering.

### 2.2. Blood Serum Collection

Whole blood serum samples from SAMP8 mice were collected by using 4.4 mL, 75 × 13 mm, Z-Gel tubes, blood allowed to clot by leaving it undisturbed at room temperature, and finally clot was removed by centrifugation at 2000× *g* for 10 min in a refrigerated centrifuge. The supernatant was collected and stored at −80 °C.

### 2.3. Blood Serum-Derived Exosomes Isolation

Serum-derived exosomes were isolated by using ExoQuick (Ref: EXOQ5A-1, System Biosciences, Palo Alto, CA, USA). The procedure was carried out by following the manufacturer’s indications. Serum was centrifuged at 3000× *g* for 15 min to remove cells and debris and the supernatant was collected. ExoQuick solution was then mixed with the supernatant and incubated at 4 °C for 30 min. ExoQuick/Serum mixture was centrifuged at 1500× *g* for 30 min. Pellet was resuspended in saline solution and stored at −80 °C for further experimentation.

### 2.4. Western Blotting Analysis

For western blotting assays, blood serum samples or isolated exosomes (30 μg of protein) were mixed with loading buffer containing 0.125 M Tris (pH 6.8), 20% glycerol, 10% β-mercaptoethanol, 4% SDS and 0.002% bromophenol blue, and heated at 65 °C for 5 min. Protein was electrophoresed on a 10% SDS-PAGE gel using a mini-protean system (Bio-Rad, Madrid, Spain) with molecular weight standards (Bio-Rad). Protein transfer to nitrocellulose membranes was carried out in iBlot ^TM^ Dry Blotting System (Invitrogen, Madrid, Spain). Membranes were washed with PBS-Tween 20, blocked with PBS containing 5% skimmed milk, and then incubated with the primary antibodies at 4 °C overnight at 1:1000 dilution for anti-A_2A_R (Abcam, ab79714), anti-A_1_R (Abcam, ab124780, Cambridge, UK), anti-mGluR_5_ (GeneTex, GTX133288, Taiwan, R.O.C.), and anti-CD9 (Santa Cruz Biotechnology, sc-13118, Dallas, TX, USA). Albumin stained with Ponceau Red was used as a loading control. After rinsing, the membranes were incubated with the corresponding secondary antibody (Bio-Rad, GAMPO 170-6516, GARPO 172-1019, Madrid, Spain) at a dilution of 1:5000 in PBS containing 5% skimmed milk for 1 h. Antigen was visualized using the ECL chemiluminescence detection kit (Amersham, Madrid, Spain) in a G: Box chamber, and specific bands were quantified by densitometry using GeneTools software (Syngene, Cambridge, UK).

### 2.5. 5′-Nucleotidase Activity Assay

5′-Nucleotidase activity was measured as previously reported [33]. Briefly, 30 μg of protein from blood serum were pre-incubated at 37 °C for 10 min in the reaction medium (50 mM Tris-HCl, 5 mM MgCl_2_ pH 9). Then, the reaction was initiated by adding AMP at the final concentration 500 μM and stopped 20 min later by adding 10% trichloroacetic acid. The samples were chilled on ice for 10 min and then centrifuged at 12,000× *g* for 4 min at 4 °C. The supernatants were used to measure inorganic phosphate released using KH_2_PO_4_ as Pi standard. The nonenzymatic hydrolysis of AMP was corrected by adding samples after trichloroacetic acid. Incubation times and protein concentration were selected in order to ensure the linearity of the reactions. All samples were run in duplicate. Enzymatic activity is expressed as nmol Pi released/min · mg protein.

### 2.6. Adenosine Level Quantification by HPLC

Chromatographic analysis was performed with Ultimate 3000 U-HPLC and data peaks were processed with Chromaleon 7 (ThermoFisher, Madrid, Spain) as previously described [17]. HPLC diode array was used working at 254 nm wavelength. Purine standards and samples (40 μL) were injected in C18 column of 4.6 mm × 250 mm, 5 μm particle size. Two solvents were used for gradient elution: solvent A 20 mM phosphate buffer solution (pH 5.7), and solvent B 100% methanol. The gradient was 95% (11 min), 80% (9 min), and 95% (2 min) in solvent A. The total run time was 22 min with a constant flow rate of 0.8 mL/min at 25 °C. The retention time for adenosine was 15.5 min. Adenosine level was obtained by interpolation from the standard curve. The standard curves were obtained by using five concentrations of adenosine ranging from 0.1–500 μM. Data were then normalized to the protein concentration of each analyzed blood serum sample.

### 2.7. Glutamate Level Quantification

The total glutamate level was quantified as indicated in the manufacturer’s protocol (Molecular Probes Ref. A12221). Briefly, 50 μL of the diluted samples were mixed into 96-black well plate with 50 μL of reaction mix containing Amplex Red, horseradish peroxidase, L-alanine and L-glutamate-pyruvate transaminase and L-glutamate oxidase. Fluorescence was measured in kinetic mode for 30 min. Data were then interpolated to a standard curve and normalized to the amount of protein. Excitation/emission was detected at Ex/Em = 530/590 nm.

### 2.8. Protein Quantification

Total protein was quantified by using the Lowry method.

### 2.9. Statistical and Data Analysis

Data are means ± SEM. Statistical analysis was according to Student’s *t*-test. Differences between mean values were considered statistically significant at *p* < 0.05. GraphPad Prism 6.0 program was used for statistical and data analysis (GraphPad Software, San Diego, CA, USA).

## 3. Results

### 3.1. Adenosine A_1_ and A_2A_ Receptors Modulation in Blood Serum

**Figure 1:**
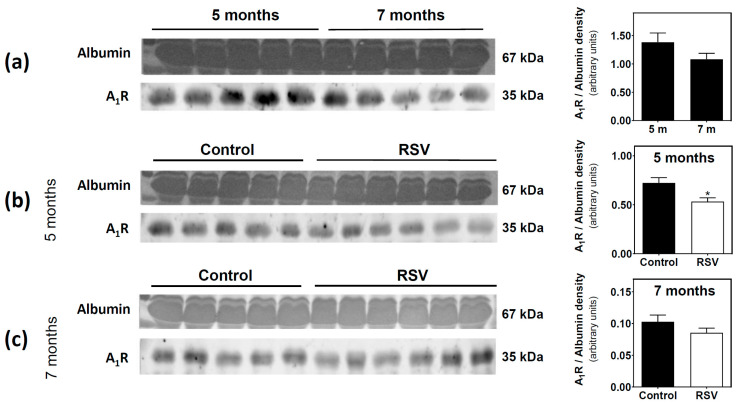
Adenosine A_1_ receptors presence and modulation in serum from SAMP8 mice. Isolated serum from SAMP8 mice was used to detect and quantify the adenosine A_1_ receptor (A_1_R) by Western blotting. (**a**) Level of A_1_R in control mice of different ages (5 and 7 months). (**b**) Effect on A_1_R levels after RSV treatment in 5 months-old mice. (**c**) Effect on A_1_R levels after RSV treatment in 7 months-old mice. Data are the mean ± SEM of five to six different samples. Albumin was used as a loading control and visualized by Ponceau red staining. * *p* < 0.05 significantly different from the corresponding control, according to the Student’s *t*-test.

**Figure 2:**
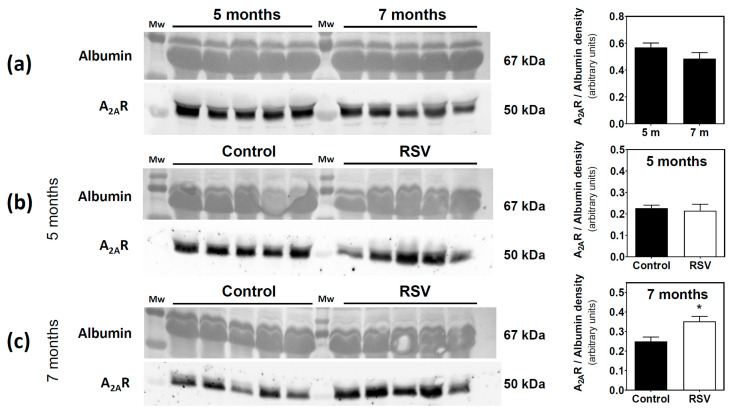
Adenosine A_2A_ receptors presence and modulation in serum from SAMP8 mice. Isolated serum from SAMP8 mice was used to detect and quantify the adenosine A_2A_ receptor (A_2A_R) by Western blotting. (**a**) Level of A_2A_R in control mice of different ages (5 and 7 months). (**b**) Effect on A_2A_R levels after RSV treatment in 5 month-old mice. (**c**) Effect on A_2A_R levels after RSV treatment in 7 month-old mice. Data are mean ± SEM of five different samples. Albumin was used as a loading control and visualized by Ponceau red staining. * *p* < 0.05 significantly different from the corresponding control, according to the Student’s *t*-test.

Adenosine A_1_ and A_2A_ receptors were detected in serum from SAMP8 mice. As shown in Figure 1, there is not a significant difference in the A_1_ receptor level between 5 and 7 mo mice (Figure 1a). However, RSV treatment caused a significant decrease in the density of this receptor in 5 mo mice (Figure 1b), whereas no changes were detected in RSV-treated 7 mo mice when compared with their corresponding untreated mice (Figure 1c). Concerning A_2A_ receptors, no changes on the level of these receptors were observed either associated with age (Figure 2a) or in 5 mo RSV-treated mice (Figure 2b), but a higher level of A_2A_ receptors was detected in RSV-treated 7 mo mice when compared with their corresponding control (Figure 2c).

### 3.2. Adenosine Level and Its Converting Enzyme in Blood Serum

**Figure 3:**
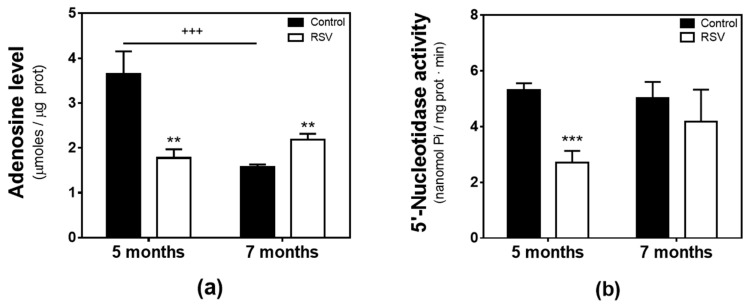
Adenosine levels and 5′-nucleotidase activity in serum from SAMP8 mice. Isolated serum from SAMP8 mice was used to measure adenosine level and its converting enzyme. (**a**) Adenosine levels and (**b**) 5′-Nucleotidase activity were quantified as described in *Methods*. Data are mean ± SEM of five-eight different samples. ** *p* < 0.01 and *** *p* < 0.001 significantly different from their corresponding controls, and +++ *p* < 0.001 significantly different from 5 months old untreated mice, according to Student’s *t*-test.

We next analyzed adenosine level and the activity of its converting enzyme, 5′-nucleotidase. Adenosine levels were found to be strongly decreased by age, as shown in Figure 3a. However, an age-dependent change on this nucleoside level was observed after RSV treatment. Accordingly, a significant decrease and increase in adenosine levels were detected in 5 and 7 mo RSV-treated mice, respectively, when compared to their age-matched controls. 5′-Nucleotidase activity (Figure 3b) did not change between 5 and 7 mo control mice, but it was significantly reduced in 5 mo mice when treated with RSV. However, this reduction was not detected in 7 mo RSV-treated mice.

### 3.3. mGlu_5_ Receptors and Glutamate Level Modulation in Blood Serum

**Figure 4:**
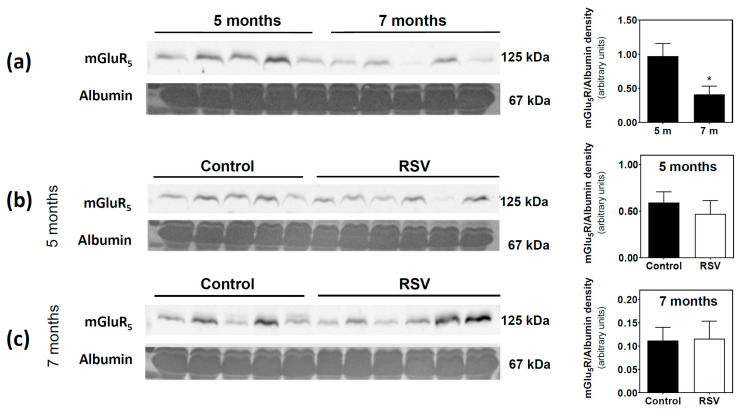
Metabotropic glutamate 5 receptors presence and modulation by RSV in serum from SAMP8 mice. Isolated serum from SAMP8 mice was used to detect and quantify the metabotropic glutamate 5 receptors (mGluR_5_) by Western blotting. (**a**) Level of mGluR_5_ in control mice of different ages (5 and 7 months). (**b**) Effect on mGluR_5_ levels after RSV treatment in 5 month-old mice. (**c**) Effect on mGluR_5_ levels after RSV treatment in 7 month-old mice. Data are mean ± SEM of four to six different samples. Albumin was used as a loading control and visualized by Ponceau red staining. * *p* < 0.05 significantly different from the corresponding control, according to the Student’s *t*-test.

**Figure 5:**
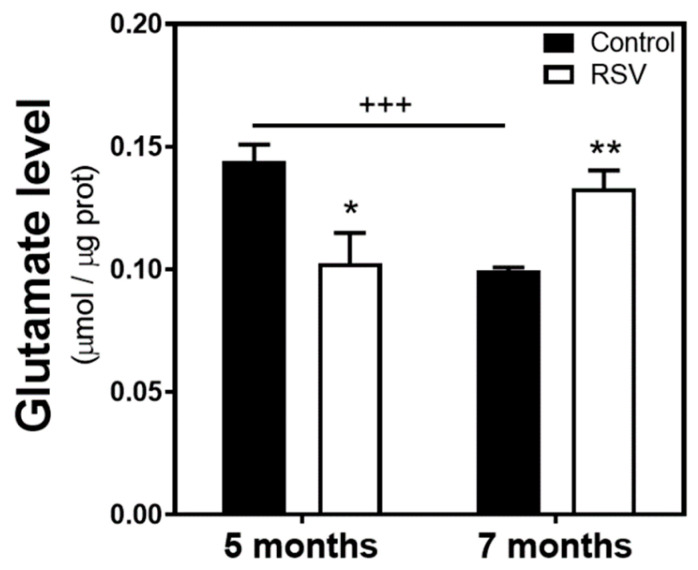
Glutamate level in serum from SAMP8 mice. Blood serum from SAMP8 mice was used to measure the glutamate level normalized to the amount of protein, as described in *Methods*. Data are mean ± SEM of five-eight different samples. * *p* < 0.05, ** *p* < 0.01 and +++ *p* < 0.001 were significantly different from their corresponding control or indicated values, according to the Student’s *t*-test.

Similarly to ARs, some components of the metabotropic glutamatergic system were detected in blood serum. Regarding mGluR_5_, a significant reduction associated with aging was observed (Figure 4a). Nevertheless, RSV treatment did not cause any effect on mGluR_5_ receptor density either 5 mo (Figure 4b) or 7 mo mice (Figure 4c). On the other hand, the glutamate level was strongly decreased by age. Yet, RSV treatment induced an age-dependent effect. A lower glutamate level was detected in 5 mo RSV-treated mice, while higher levels were found in 7 mo RSV-treated mice when compared to their corresponding controls (Figure 5). Albumin level, which has been used as a gel loading control, was quantified in the different conditions studied. The level of this protein was unchanged by age or RSV-treatment (Figure S1).

### 3.4. Adenosine A_1_ and A_2A_ and mGlu_5_ Receptors Presence in Blood Serum-Derived Exosomes

**Figure 6:**
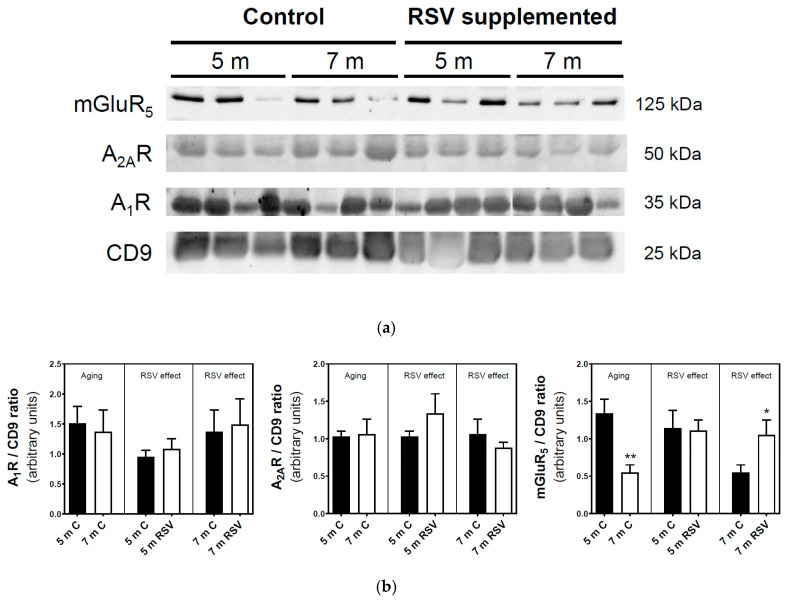
Adenosine A_1_, A_2A_ and metabotropic glutamate 5 receptors in serum-derived exosomes from SAMP8 mice. Serum-derived exosomes from SAMP8 mice were isolated following the manufacturer’s indications, and different proteins were detected by Western-blotting as described in “*Methods*”. (**a**) Representative gel bands of mGluR_5_, A_2A_R, A_1_R, and CD9 presence in exosomes. (**b**) Level of mGluR_5_, A_2A_R, A_1_R, in control (C) and resveratrol supplemented (RSV) mice of different ages (5 and 7 months). Data are mean ± SEM of five to eight different samples. CD9 was used as a loading control. * *p* < 0.05, and ** *p* < 0.01 significantly different from the corresponding control, according to the Student’s *t*-test.

To further investigate whether these receptors can be found into circulation in exosomes, we isolated blood serum-derived exosomes by using ExoQuick, as described in *Methods*. A strong immunoreactivity against CD9, considered an exosome marker, was detected in the exosome fraction (Figure 6a). In addition, A_1_R, A_2A_R, and mGluR_5_ were also detected, and quantified in exosomes (Figure 6b).

## 4. Discussion

Results presented herein show, for the first time, the presence of ARs and mGluRs in blood serum and exosomes, as well as their modulation by aging and RSV supplementation. Furthermore, adenosine and glutamate levels were also modulated by age and RSV supplementation.

SAMP8 mice have been considered an aging and an AD model. Accordingly, it has been reported similarities to the pathophysiology of aging in the human brain and the early cognitive decline [34] together with other distinctive hallmarks of AD such as Aβ overexpression, upregulation of Presenilin-2 and high levels of p-Tau in the hippocampus, but lower expression of Apolipoprotein-E as compared to their respective control mice [35]. The lifespan for SAMP8 is around 10 months of age [36]. According to the half lifespan of a common mice strain and the maturational rates mouse vs. human, 2 mo represents a young human, and 4 mo a middle-aged individual, when our RSV treatment starts. We evaluated SAMP8 mice at 5 (middle aged, 38–47 years) and 7 months (old individual, 56–69 years) [37].

The modulation of ARs and mGluRs has been reported in different brain areas of AD patients [18,23]. In the whole-brain from SAMP8 mice, we have reported an age-related downregulation and desensitization of the A_1_ receptor whereas A_2A_ was found to be fully functional [29,38]. Also mGluR_5_ significantly decreased with aging [30]. These previous data suggest SAMP8 mice as a suitable model for ARs and mGluRs related research on neurodegenerative diseases.

Now, our results indicate that A_1_, A_2A_, and mGlu_5_ receptors are present in blood serum from SAMP8 mice. These receptors could be released into circulation likely via exosomes since they were detected in blood serum-derived exosome as well as CD9, a tetraspanin widely used as exosome marker [39,40,41]. The presence of different GPCRs in blood serum has been evidenced before. Corticotropin releasing-factor receptors I/II (CRF receptor I/II) were reported as circulating receptors in extracellular vesicles (EVs) from blood serum [32]. Additionally, the purinergic receptor P2 × 7 was found as EVs cargo in human blood serum. Although P2 × 7 receptors were identified as a full-length molecule, some bands with lower molecular weight were also detected. In fact, the authors suggested that proteolytic cleavage could not be excluded from shedding into the circulation of this receptor [31]. We detected circulating A_1_R at 35 kDa when in brain tissue it was detected at 37 kDa. This discrete but lower molecular weight of circulating receptors found in serum when compared to the brain receptors could be related to a proteolytic cleavage during the releasing process. However, brain A_2A_R can be detected at 45 kDa, but circulating A_2A_R was detected at 50 kDa. The higher molecular weight observed in this receptor could be due to glycosylation or related-mechanism likely to facilitate their transport in blood serum. In accordance, circulating CRF receptor I/II were also detected at a discrete but higher molecular weight in human blood serum [32]. Regarding mGluR_5_, it was found a band at 125–130 kDa, which is in line with the predicted weight estimated by the manufacturer’s indications, suggesting that this receptor might be released as a full-length molecule.

Some authors found β-actin in plasma and not significant changes in its density were observed in major depressive disorder (MDD), thus allowing their use as a loading control for plasma-based Western blotting [42]. However, we found some density changes associated with age in SAMP8 serum, as previously reported in human skeletal muscle cells [43]. Therefore, we instead used albumin, the most abundant protein in serum, as a loading control. It has been postulated a connection between dementia and blood-brain barrier (BBB) dysfunction [44], which could lead to altered CSF/serum albumin index due to the BBB disruption [45]. Here, we did not found changes in albumin density either associated with age or RSV supplementation (Supplemental Figure S1).

It is widely known that both adenosine A_1_ and A_2A_ receptors [46] and their endogenous ligand [47] are unevenly distributed throughout the healthy human brain. Adenosine A_1_ receptor is the most abundant subtype within the CNS except for the striatum, putamen, and basal ganglia, where the A_2A_ receptor is highly abundant [48]. This uneven expression of ARs within the CNS is accompanied by differential ARs modulation in each brain area of AD patients. A widespread lower level of A_1_ receptors in AD patients as compared to healthy individuals was observed by PET [49]. Similarly, an age-related loss of this receptor in the whole-brain of SAMP8 mice was also described [29,38]. In contrast, an increased density of A_1_ receptors was detected in the frontal cortex from the *post-mortem* human brain of AD patients [18]. On the other hand, it was not found a clear alteration on the A_2A_ receptors density detected by PET during aging in the human brain [50]. These results are in line with a previous work where no changes were found on the A_2A_ receptors density in plasma membrane from the whole-brain in SAMP8 mice during aging [29]. However, it has been reported a significantly increased density of A_2A_ receptors in the limbic cortex but not in the striatum in aged rats [51], as well as an up-regulation of A_2A_ receptors in the frontal cortex from post-mortem brain of AD patients [18].

Regarding adenosine levels, it has been described a different pattern of distribution and modulation of this nucleoside together with the activity of its converting enzymes in several areas from the human brain cortex of AD, even at the early stages of the disease, as compared to healthy controls [19]. Due to area, age, and gender dependence of the nucleoside system in the brain [52,53], it is difficult to conclude how adenosine level is modulated in the whole-brain from AD patients. The lack of data about a global change in adenosine level in the whole brain in AD avoids its possible correlation with the increased adenosine levels reported in serum [54]. However, we found an age-related decrease of adenosine in SAMP8 serum, associated with a reduced level in the whole-brain of these mice [29]. In humans, the quantification of plasma adenosine concentration in 1141 patients revealed that advancing age may be associated with lower adenosine levels [55]. The reported gradual increase in the activity of serum adenosine deaminase could be a contributing factor [56].

An interesting but less investigated enzyme in AD is the 5′-Nucleotidase activity. A previous study demonstrated a significant decrease in this enzymatic activity in the frontal cortex of AD patients as compared to age-matched healthy controls [19]. This activity was also decreased in the whole-brain of aged SAMP8 mice [29]. However, the absence of changes related to age on the 5′-Nucleotidase activity in blood serum, besides a dramatically lower activity in serum than in the brain [29], makes it difficult to establish an association between serum and brain enzymatic activities.

The pathological role of mGluR_5_ in the CNS has been the focus of intense research since a direct interaction of Aβ and mGluR_5_ was reported [22,57]. mGluR_5_ plays a crucial role in the cognitive decline, and it could be involved in the pathogenesis and progression of AD [58,59]. However, little is known about the modulation of this receptor in the brain from AD patients. Previous work reported an absence of changes in the mGlu_5_ density in the frontal cortex from *post-mortem* samples of AD patients, despite an impaired functionality of group I mGluRs observed even at early stages [23]. However, an in vivo study by PET revealed a downregulation of mGluR_5_ caused by Aβ in the limbic system in the 5xFAD mouse model as compared to wild type [60]. We have recently described a significant age–associated decrease in mGluR_5_ density in the whole-brain of SAMP8 mice [30]. Interestingly, in the present work, a significant and robust reduction in mGluR_5_ density was also detected in blood serum and exosomal fraction from 5 to 7-month-old.

It has been reported that synaptic glutamate level shows a tendency to increase in AD [20,61], which can lead to excitotoxicity and neuronal death [21]. Other authors revealed a decreased level of glutamine in serum from AD patients, suggesting that glutamate metabolism could be altered [54].

An age-related reduction in glutamate levels in the whole-brain of SAMP8 mice from 5 to 7 mo mice was reported [30], which is in agreement with the decrease in glutamate content in the cerebral cortex and hippocampus from SAMP8 mice monitored from 2 to 14 mo animals [62]. Interestingly, a similar and significant reduction of glutamate levels is now reported in serum from 5 to 7 mo mice. In healthy humans, serum glutamate level was not significantly different between 38–47 and 56–69 years old [63], which is equivalent to 5 and 7 mo SAMP8 mice. Interestingly, serum levels of glutamate progressively decreased from healthy subjects over mild cognitive impairment to AD [64]. Therefore, the decrease in serum glutamate reported here could represent the progression of the disease from 5 to 7 mo mice in this model of AD. Brain-to-blood efflux of glutamate occurs through the blood-brain-barrier [65]. Thus, serum glutamate levels is the result of glutamate originated in blood cells and peripheral organs, and its efflux from the brain [66].

Additionally, RSV supplementation caused an age-dependent modulation in serum glutamate levels, which is in line with the neuroprotective effect exhibited by this polyphenol. In C57BL/6J mice, oral administration of RSV results in a maximal plasma concentration (C_max_) of ~12 µM for 100 mg/kg b.w. [67], and ~32 µM for 240 mg/kg b.w. [68]. In 5-month-old SAMP8 mice, we have detected a serum RSV concentration of 0.044 μM after oral administration of 120 mg/kg b.w. for 8 weeks. This RSV level is not a C_max_ value but the concentration found in serum when mice were sacrificed [69]. Taking into account that RSV acts as a non-selective ARs agonist [70], and these receptors can fine-tune the physiological activity of mGluRs [71], it is conceivable that the in vivo modulation of ARs in the brain from SAMP8 mice [29] might be responsible, at least in part, for the modulation of the glutamatergic system [30].

One interesting finding is the similarity of changes on the levels of ARs and mGluRs and their endogenous ligands (i.e., adenosine and glutamate) when comparing brain and serum derived results. Table 1 summarizes data obtained in the present work (i.e., blood serum) with that previously reported by our group concerning the adenosinergic [29] and the glutamatergic [30] signaling in the whole-brain of SAMP8 mice. Thus, adenosine, glutamate, and mGluR_5_ are significantly and similarly decreased in serum and whole-brain during aging and in RSV treated mice of 5 months of age. A_2A_R levels seem to be preserved in both serum and whole brain during aging or RSV supplementation. However, changes in adenosine A_1_ receptors are more erratic, and it cannot be established a clear correlation between serum and whole brain values. This correspondence between serum and whole brain obtained values could be a promising discovery in the development of new and feasible biomarkers in AD.

To date, many studies have aimed to decipher whether distinctive hallmarks of AD present in serum such as oxidative stress, mitochondrial dysfunction, high expression of pro-inflammatory cytokines [10], Aβ deposition [11] and p-Tau [72,73] have a predictive value in early diagnosis of AD [9]. Unfortunately, the weak correlation between CSF and plasma Tau together with the wide variability of Aβ levels reported in blood confer to these main hallmarks of AD a poor predictive and diagnosis value, suggesting the need for more accurate AD biomarkers. Fortunately, some other molecules present in peripheral blood such as neurotransmitter (e.g., glutamate, adenosine) [54] and related-receptors, cholesterol [74] or iron [75] that have been reported to be altered in AD could be used in combination with classical markers as potential blood-based biomarkers to aid in early diagnosis of AD in the future. In addition, the analysis of ARs and mGluRs in blood serum could be the basis of new biomarkers development in the context of AD.

Western blotting quantification of circulating receptors could be impractical for future clinical applications since this technique does not provide an absolute but relative quantification. Methodologies such as radioligand binding assay could be the ideal candidate to quantify circulating receptors in blood serum due to its high sensibility and absolute quantification. Nevertheless, we unsuccessfully tried to quantify A_1_R, A_2A_R, and mGluR_5_ by using this method. Probably, the high abundance of albumin, which represents about 50% of the total protein content in blood serum samples, was interfering in our assays. Transport of molecules is one of the main biological functions of albumin. In agreement, we observed a radioligand uptake by albumin alone, which interfered with the radioligand binding assay leading to not reliable results.

The biological significance of the presence of plasma membrane-receptors as extracellular vesicle cargo in blood serum has not been elucidated yet. This phenomenon could be involved in cell-to-cell communication and the regulation of GPCRs [76]. In fact, it has been reported in vitro that A_1_R, A_2A_R, and A_2B_R participate in modulating exosome production by cells expressing these receptors [77]. Despite the well-known molecular mechanisms by which GPCRs are desensitized and internalized in a cell, there is a lack of knowledge on how these receptors can be released into circulation or what types of stimuli may trigger their secretion. Our study opens new possibilities on how GPCRs might be modulated not only through desensitization and internalization but also by secreting receptors into circulation and later uptake by other cells, in a process where exosomes or extracellular vesicles seem to have a role. Future studies are required to delve into the biological significance of these findings.

## 5. Conclusions

Our data show: (i) evidence of the presence in serum and exosomes of some GPCRs such as A_1_R, A_2A_R, and mGluR_5_, (ii) its modulation by aging and resveratrol, and (iii) a potential association between brain and serum receptors levels. Even though further investigations are required to find out whether this association can also be found in humans, or to assess the origin of these receptors (e.g., are they brain-derived?), we suggest that the detection of these receptors in blood serum and exosomes would merit attention in the research of early diagnosis of AD.

## Figures and Tables

**Table 1:** Summary of changes detected in blood serum and whole-brain of SAMP8 mice.

	AGING		RSV Supplementation			
	(from 5 to 7 Month-Old)		5 Month-Old		7 Month-Old	
Parameter	Serum	Brain	Serum	Brain	Serum	Brain
A_1_R	↓ 22%, ns	↓ 64%, **	↓ 26%, #	↑ 46%, ##	=	↑ 309%, ###
A_2A_R	**=**	**=**	**=**	**=**	↑ 41%, #	=
mGluR_5_	↓ 58%, *	↓ 44%, ***	=	=	=	=
Adenosine	↓ 57%, ***	↓ 59%, **	↓ 52%, ##	↓ 39%, #	↑ 38%, ##	=
Glutamate	↓ 29%, ***	↓ 23%, *	↓ 29%, #	↓ 27%, #	↑ 31%, ##	↓ 14%, #

The percentage of increase (↑) or decrease (↓) detected on each parameter when comparing 7- versus 5-month-old control animals (*Aging*) or RSV treated versus corresponding control animals (*RSV supplementation*) on blood serum and whole-brain of SAMP8 mice. Similar changes are indicated in bold. ns, not significant. * *p* < 0.05, ** *p* < 0.01, *** *p* < 0.001 significantly different from 5 months-old control animals. # *p* < 0.05, ## *p* < 0.01, ### *p* < 0.001 significantly different from corresponding control animals.

## Supplementary Materials

The following are available online at https://www.mdpi.com/2073-4409/9/7/1628/s1, Scheme S1: Resveratrol treatment schedule. Figure S1: Ponceau red staining of electrophoresed proteins.
