# Supplementary material for: Adenosine and Metabotropic Glutamate Receptors Are Present in Blood Serum and Exosomes from SAMP8 Mice: Modulation by Aging and Resveratrol
Source: Cells. 2020 Jul 7;9(7):1628. doi: 10.3390/cells9071628 (PMC7407497; doi:10.3390/cells9071628)
Supplement: Supplementary file 1 [file cells-09-01628-s001.zip › supplemental figure 1.pdf]

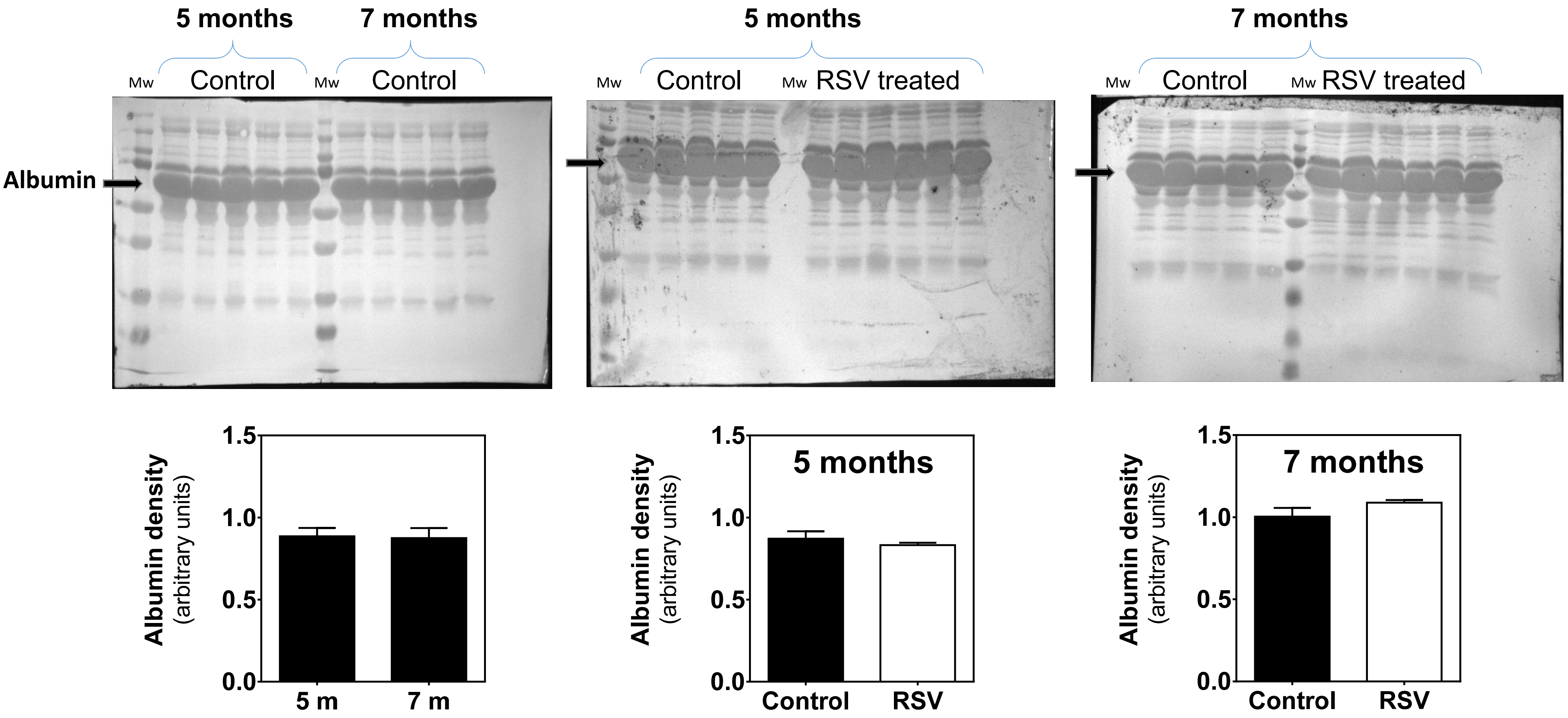

Supplemental figure 1. Ponceau red staining of electrophoresed proteins. Albumin remained unchanged in all experimental groups.
