# Supplementary figures and images for: Adenosine and Metabotropic Glutamate Receptors Are Present in Blood Serum and Exosomes from SAMP8 Mice: Modulation by Aging and Resveratrol
Source: Cells. 2020 Jul 7;9(7):1628. doi: 10.3390/cells9071628 (PMC7407497; doi:10.3390/cells9071628)

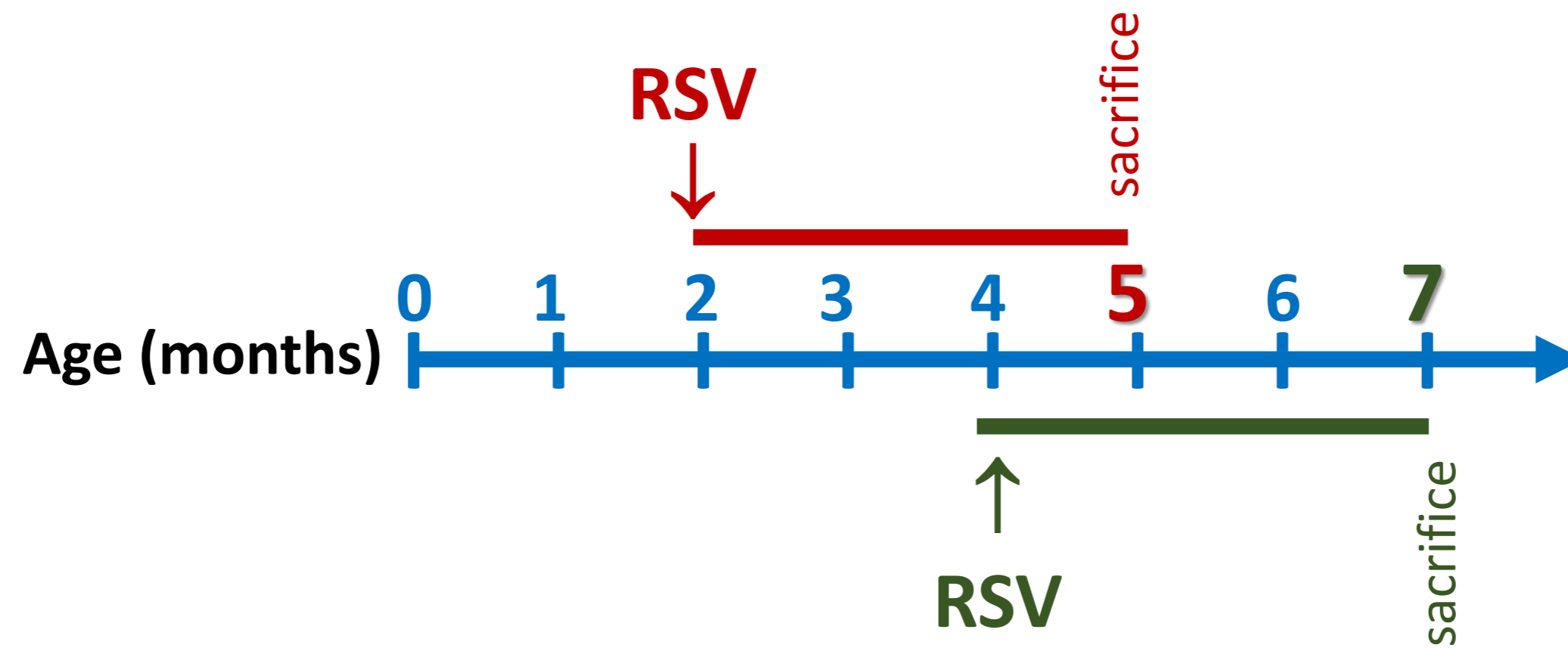

Scheme 1. Resveratrol (RSV) treatment schedule followed in the present work.

Supplement: Supplementary file 1 [file cells-09-01628-s001.zip › scheme 1 SERUM.pdf]
